# Effect of unfocused extracorporeal shockwave therapy on bone mineral content of twelve distal forearms of postmenopausal women: a clinical pilot study

**DOI:** 10.1007/s11657-019-0650-x

**Published:** 2019-11-26

**Authors:** Marianne K. E. Koolen, Moyo C. Kruyt, Fetullah C. Öner, Wolfgang Schaden, Harrie Weinans, Olav P. van der Jagt

**Affiliations:** 1 Department of Orthopaedics, University Medical Centre Utrecht, Utrecht, The Netherlands; 2 Department of Traumatology, AUVA Trauma Center Meidling, Vienna, Austria; 3 Department of Rheumatology and Clinical Immunology, University Medical Centre Utrecht, Utrecht, The Netherlands; 4 Department of Biomechanical Engineering, Faculty of Mechanical, Maritime, and Materials Engineering, Delft University of Technology, Delft, The Netherlands; 5 Department of Orthopaedics, Elisabeth-TweeSteden Hospital, Tilburg, The Netherlands

**Keywords:** High-energy shockwaves, Bone density, Osteoporosis

## Abstract

***Summary*:**

Extracorporeal shockwave therapy showed a pronounced effect on bone mass in previous animal studies. We showed in this pilot study that a single treatment with unfocused shockwave therapy in unselected patients does not show side effects. Although our study did not show any effect of shockwave on BMD, the limited sample size does not definitively exclude this and a study with 174 subjects per group would be needed to show an effect size of 0.3 with a power of 80%.

**Purpose:**

Unfocused extracorporeal shockwave therapy might stimulate bone formation to reduce the fracture risk. In this study, we assessed the safety of unfocused extracorporeal shockwave therapy and its effects on bone mass.

**Methods:**

A clinical pilot study with twelve female patients free of bone disease undergoing elective surgery of the lower extremity or elective spinal surgery under general anesthesia received 3.000 electrohydraulic-generated unfocused extracorporeal shockwaves (energy flux density 0.3 mJ/mm^2^) to one distal forearm. The contralateral forearm served as a control. We examined the effect on bone mass with the use of repeated dual energy X-ray absorptiometry measurements and we measured patient discomfort around the therapy.

**Results:**

No difference in bone mineral content and density was measured 6 and 12 weeks after therapy. shockwave therapy occasionally caused transient erythema or mild hematoma, but no discomfort in daily life or (late) adverse events.

**Conclusions:**

Unfocused extracorporeal shockwave therapy is a safe treatment, but no increase in bone mass on the forearm was found at 0.3 mJ/mm^2^ energy flux density. In this study, we were not able to demonstrate that a single treatment with unfocused shockwave therapy in unselected patients had any effect in terms of bone mineral density (BMD) or bone mineral content (BMC). A power analysis indicated that 174 patients per group are required to show an effect size of 0.3 with a power of 80%.

## Introduction

Osteoporosis is a disease characterized by bone loss and deterioration of the bone micro architecture, leading to a higher susceptibility for fractures. These fractures have a severe impact on patient’s well-being [1–3].

In order to reduce osteoporosis-related mortality and morbidity, fracture prevention and early diagnosis are the primary goals [4]. Today’s standard treatment is lifestyle modifications, supplementation of calcium and vitamin D in combination with bisphosphonates, which reduce osteoclastic driven bone resorption [5]. Although these drugs are effective, most have some limitations and side effects that affect long-term administration and adherence [6, 7].

In the search for an alternative, preferably anabolic therapy, we examined the application of extracorporeal shockwaves (ESW), which showed a pronounced effect on bone mass, leading to relatively early improved biomechanical properties in previous studies in the rat tibia [8–11].

Shockwaves are acoustical pulses that are characterized by high amplitude (~ 500 bar) and short rise time (~ 20 ns), which are followed by a longer low-magnitude negative wave (~ − 100 bar) [12]. ESW are widely used to disintegrate kidney stones [13]. In orthopedics, shockwave therapy is used safely and effectively in a variety of musculoskeletal disorders like non-unions, osteonecrosis of the hip and tendinopathies [14–17]. Until recently, extracorporeal shockwave therapy for musculoskeletal disorders was applied focused, i.e., the waves converge in a focal point [18]. For the prevention of fractures in osteoporosis, a focused approach is not preferable because relatively large skeletal regions have to be treated. For the treatment of dermatologic pathologies, generators that produce unfocused shockwaves have been developed, which produce a parallel bundle, enabling a homogenous treatment of larger areas [19]. Skeletal sites, such as the distal radius and proximal femur, are particularly interesting candidates to examine, because they represent well-known locations of osteoporotic fractures and are easy to reach for shockwave therapy [20]. When indeed bone density increase can be achieved at clinically relevant levels, this may be a non-invasive additive to today’s osteoporosis treatment or locally improve bone quality for better osteosynthesis. In the current clinical pilot study, the safety as well as potential magnitude and duration of the anabolic effects of unfocussed shockwave therapy is therefore being assessed in the distal forearm of twelve patients.

## Methods

### General

We designed this phase II, randomized, single-blind, intervention study with an internal control to assess safety and efficacy of a single-time shockwave therapy to increase bone density. The study was conducted at one institution with twelve patients treated between May 18, 2015, and September 19, 2016.

### Participants

Eligible patients were females, age 50–80 years, on the list for elective surgery of the lower extremity or spine under general anesthesia. Since this study only assessed a general effect on bone density, we did not select osteoporotic patients. They should have a normal dietary intake inclusive calcium and/or milk products. Exclusion criteria were skin disease, systemic corticosteroid use, known systemic disease that interacts with bone (e.g., rheumatoid arthritis, multiple myeloma, hyper(para)thyroidism, Paget’s disease, or Cushing’s disease), or a previous wrist fracture.

### Ethics

The study protocol was approved by the Ethics Committee (02 April 2014, MEC 2012-453 | NL40580.078.12) and was registered in the ClinicalTrials.gov Protocol Registration System (NCT02630381). Before enrollment, written informed consent was obtained from each participant included in the study.

### Study procedures

**Fig. 1:**
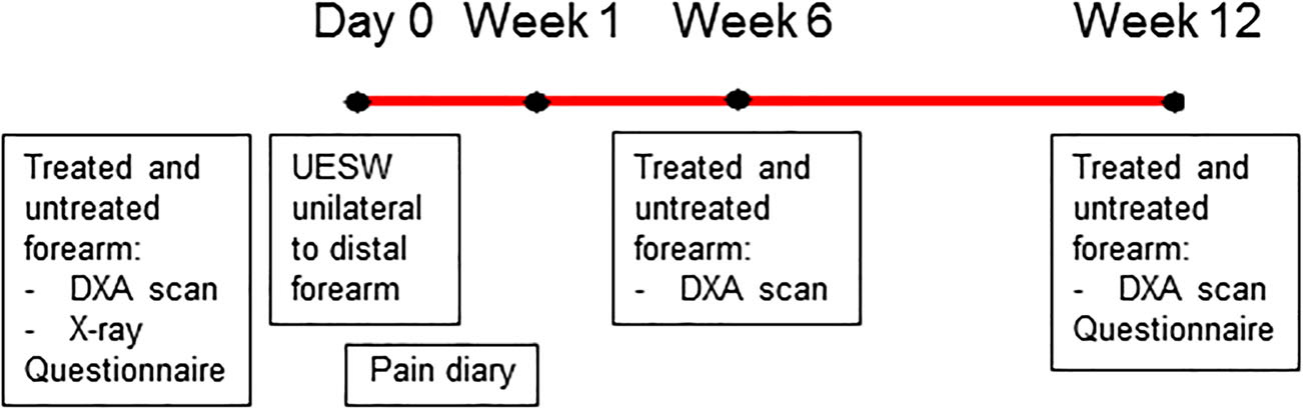
Timeline with procedures that subjects will undergo in the course of the study. UESW, unfocused extracorporeal shockwave therapy

After informed consent, twelve patients were included. Baseline measurements were obtained including a DXA-scan of both wrists and patients were randomized to receive perioperative shockwave therapy to the left or right distal forearm. The forearm was chosen as it is a prone site for an osteoporotic fracture. On top of that, it is easily reachable and there is an internal control. One independent person had access to the computer-generated randomization lists. The patients were blinded for which arm was treated. The distal forearm that did not receive unfocused extracorporeal shockwave therapy was used as a control. Follow-up measurements were planned and performed during the first week in the hospital or at home in a diary and in the hospital at the 6th and 12th week after UESW therapy (Fig. 1).

### Unfocused extracorporeal shockwave therapy

Directly after general anesthesia, one arm was treated with 3.000 unfocused, electrohydraulic-generated shockwaves (UESW) with a treatment area of 3.8 cm in diameter, with an Orthogold 180c (MTS Medical, Konstanz, Germany). The shockwaves were applied around the distal forearm with an energy flux density of 0.3 mJ/mm^2^, with a frequency of 5 Hz. An ultrasonic gel (Aquasonic, Parker Laboratories, Almelo, The Netherlands) was applied as coupling media between the applicator and the skin on the side that was going to be treated. This energy flux density was chosen by literature based on non-union treatment and experience of one of the co-authors [21, 22]. The applicator was moved 180° around the dorsal distal forearm to make sure all bone interacted with the shockwave-related energy. After the therapy, the gel was removed. The contralateral arm served as a control and was not treated.

### Study parameters

Before treatment, we gathered baseline characteristics with a questionnaire and by consultation. Side effects, adverse events, medical consumption (visits to health care providers and consumptions of prescript or over the counter medication), and nuisance in daily activities were evaluated with a questionnaire at the last appointment, and we also asked them which forearm they thought was treated.

### Radiographic assessment

**Fig. 2:**
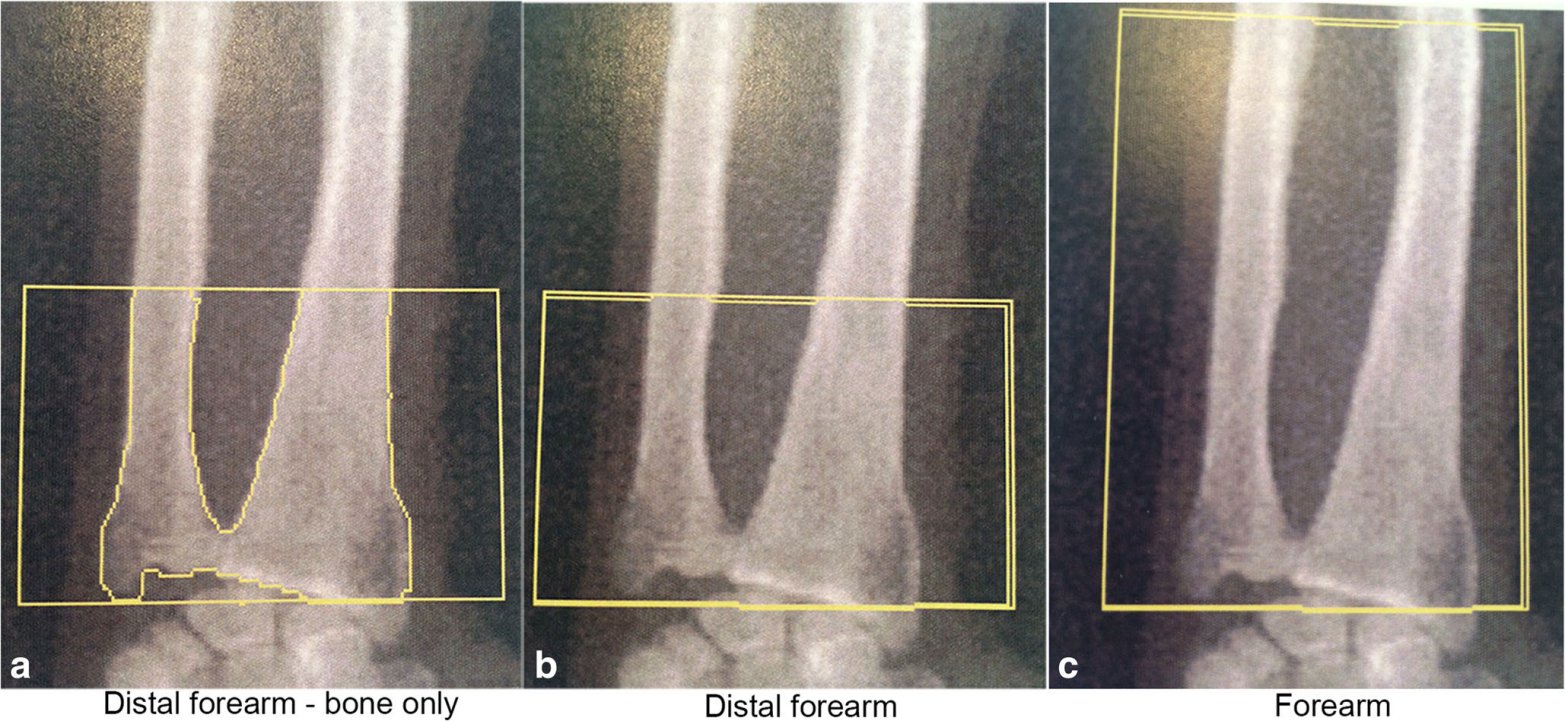
DXA-scans with areas of analysis

An X-ray of both distal forearms was made to identify pre-existent disorders. A DXA-scan (Hologic Discovery A DEXA, S/N 80675) to determine bone mineral content (BMC, grams) and bone mineral density (BMD, grams per square centimeter; g/cm^2^) in three areas of both distal forearms at baseline and after UESW treatment was our primary outcome measure. The three areas were as follow: (A) directly around the bone in the distal forearm (distal forearm—bone only); (B) a fixed square around the bone of the distal forearm (distal forearm); (C) a fixed square around the forearm (forearm) (Fig. 2). All areas were referenced to the distal ulnar styloid process. Also, a *T* score at baseline was measured for the total forearm according to standardized DXA-scan procedures according the Hologic database. Follow-up DXA-scans were performed at 6 and 12 weeks after the shockwave treatment. Positioning over time was the same in each patient. Scans and scan analysis were performed by one experienced independent person blinded to which side was treated.

### Safety and clinical assessment

Pain at the distal forearms was determined with a visual analogue scale (VAS) before the operation and during the first week with a diary (three times daily). We performed clinical and physical examination of the distal forearms when the patient was sufficiently recovered from the operation. In case of pain at the distal forearm, a standard pain protocol was followed.

### Statistical analysis

The data are presented as means and standard deviation unless otherwise indicated. In the analysis of the results of the DXA-scanning (BMD and BMC in three different areas), a mixed model was used to test for statistically significant differences between the UESW-treated and control distal forearm, while correcting for time effect and for paired effect using subject and distal forearm (SPSS 21.0 software IBM, Armonk, NY, USA). The analysis of the VAS was also performed with a mixed model to test for statistically significant differences between the UESW-treated and control distal forearm, while correcting for subject and distal forearm. A *p* value less than 0.05 was considered to indicate a statistically significant difference. Based on these pilot data, we performed a sample size calculation to determine the required sample to confirm efficacy of UESW with 80% power (https://clincalc.com/). For that purpose, a clinical relevant effect of 0.3 SD difference in bone mineral density on DXA of the bone directly around the distal forearm was assumed [23–25].

## Results

### Demographics/study parameters

All patients completed the study. The following operations were performed: spine (58.3%; 6 (re)spondylodesis and 1 removal of osteosynthesis), hip (25%; 1 total hip arthroplasty, 1 cup, and 1 stem revision of hip arthroplasty), and knee (16.7%; 1 proximal tibial osteotomy and 1 knee distraction) surgery. At the last appointment, we asked the patients if they knew which distal forearm was treated. Three patients thought they were treated at the untreated distal forearm, two patients made the right assumption, and seven patients did not know which distal forearm was treated.

At the time of operation and UESW treatment, the mean age of the patients was 57 (range 50–75) and the mean weight and height were 76.5 kg (range 54–134) and 167.5 cm (range 160–178). All patients were from a Dutch origin and living in the Netherlands; in 91.7%, the dominant side was right. There was no abnormal daily activity mentioned by any of the patients. There were no patients with a musculoskeletal co-morbidity beside the primary diagnosis for which they were operated. One person smoked, and another person used more than two units of alcohol daily. No one had a history of parental hip fracture or known osteoporosis.

### Radiographic evaluation

All scans could be analyzed. The average time between the first DXA-scan and the treatment was 5.4 weeks (SD 5.6). The time between the treatment and the first post-treatment DXA-scan was 6.4 weeks (SD 1.0), and to the second post-treatment, DXA-scan was 12.8 weeks (SD 1.1). The baseline DXA showed a *T* score of − 0.73 (SD 1.01) in the distal forearm DXA-scan analysis. The average bone mineral density and bone mineral content in the three areas at baseline were respectively 0.45 g/cm^2^ (SD 0.07) and 7.61 g (SD 1.11) (A), 0.21 g/cm^2^ (SD 0.03) and 8.02 g (SD 1.25) (B), and 0.24 g/cm^2^ (SD 0.026) and 17.02 g (SD 1.84) (C).

**Fig. 3:**
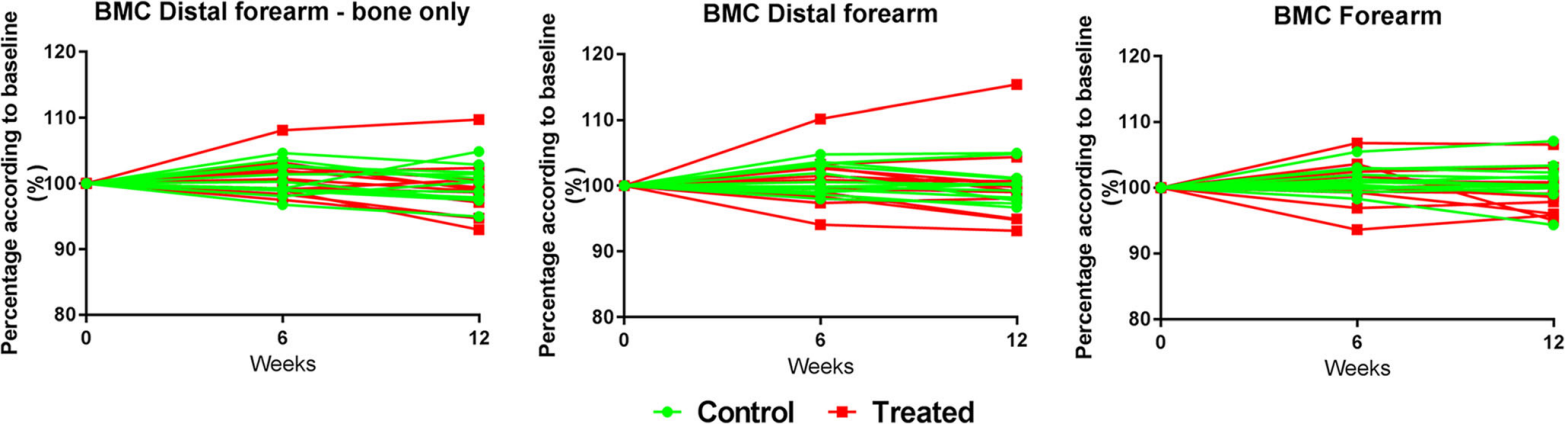
Radiographic evaluation with DXA-scanning of BMC in three different areas

There was no difference in BMC and BMD between treated and control distal forearms, in any of the analyzed areas (Fig. 3a; *p* = 0.840, Fig. 3b; *p* = 0.820, Fig. 3c; *p* = 0.845). The average BMC at 12 weeks after treatment in the treated forearms compared with baseline was respectively 98.1% (SD 5.6) at the “distal forearm—bone only” readout, 100.1% (SD 6.0) at the “distal forearm” readout, and 99.4% (SD 3.2) at the “distal forearm” readout. In the untreated forearms, this was respectively 99.9% (SD 2.7), 100.2% (SD 2.7), and 100.9% (SD 3.0). There was one patient who had a clear increase in BMC (Fig. 3). In this patient, at the “distal forearm—bone only” readout (Fig. 2a), there was an increase in BMC of 9.8%, at the “distal forearm” readout (Fig. 2b), the increase in BMC was 15.4%, and at the “forearm” readout (Fig. 2c), the increase in BMC was 6.6%. This was the patient with the lowest BMC at baseline (− 2.2 g). Other baseline characteristics or questionnaires did not show anything abnormal.

Post hoc analysis was performed to determine the sample size needed to be able to demonstrate efficacy of UESW in BMD between untreated and shockwave-treated forearms with 80% power. We assumed 0.3 SD as the non-inferiority margin and used the group means and SD of 0.06 g/cm^2^ from our study for this analysis, resulting in a sample size of 174 patients per treatment group. As we used an internal (paired) control in our group (i.e., treated versus untreated forearms within humans), this number is a conservative estimate.

### Safety and clinical outcomes

**Fig. 4:**
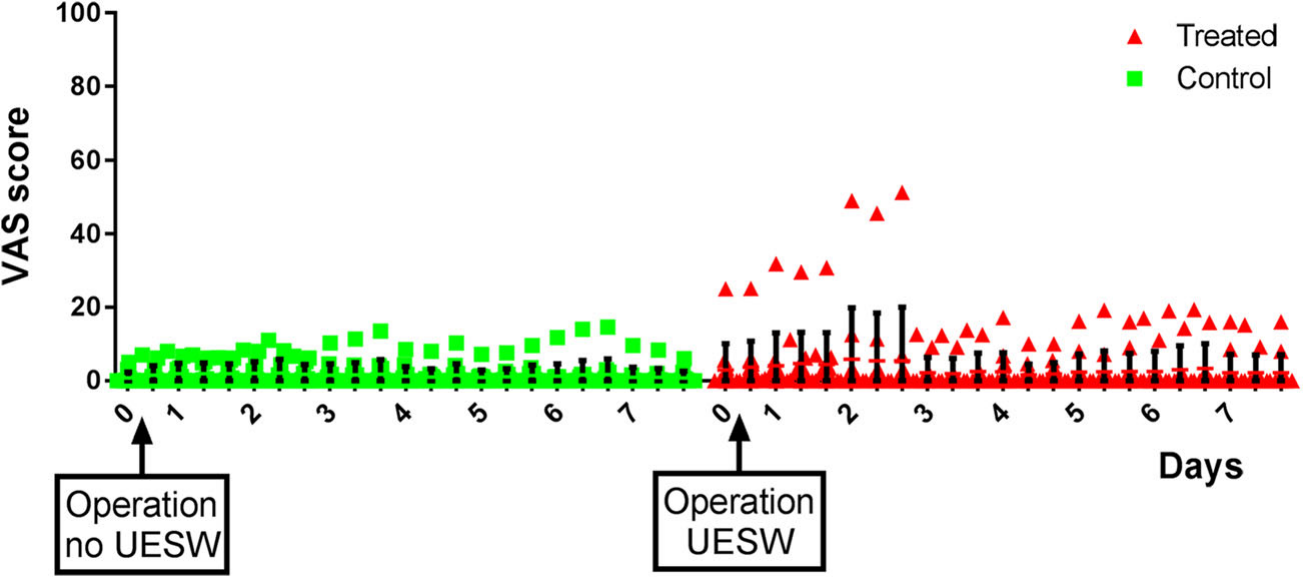
VAS score of each person at each time point of the pain diary. UESW, unfocused extracorporeal shockwave therapy

VAS score was postoperatively different if treated and control arms were compared (Fig. 4; *p* < 0.001), which was due to one patient. This patient already had pain complaints preoperatively on the side that was treated. If the VAS scores are compared with preoperatively, no difference was noted between the treated and untreated arm in the first week (both parametric metric and non-parametric test showed *p* = 0.96). No X-rays were made after UESW therapy, as there were no clinical and physical signs of a fracture. No complaints were reported after treatment, neither did any of the patients use pain killers for pain in their forearms. However, one patient reported a higher VAS score in the treated forearm. This patient showed a small increase in the BMC of the “distal forearm—bone only” area of 0.6% after 12 weeks. Other measured values did not show an increase, neither a decrease. Two patients had redness and two others had a hematoma of the treated distal forearm 1 day after the treatment. One patient noticed redness of the untreated distal forearm and another pressure pain of the untreated distal forearm, from unknown origin. No other medical consumption, nuisance in daily activity, was mentioned by any of the patients except for what was expected due to the operation. The postoperative medication, including pain medication, was given according to our standard protocols.

## Discussion

In this pilot study, we showed that application of unfocused perioperative shockwave is feasible and without adverse events. However, no effect in terms of bone density increase or bone area could be observed in the forearm based on any of the regions of interest analyzed. Many studies investigated the effects of ESW therapy in animal experiments on bone [8, 9, 26, 27] and in humans on non-unions [21, 22, 28–30]. Although previous animal experiments reported positive results, in the current study, no effect of shockwave therapy could be demonstrated. It might be that the anabolic effect was already diminished 6 weeks after the UESW treatment. However, since bone turnover and bone resorption processes are rather slow, this is not a likely option. In addition, if the increase is not sustained for more than 6 weeks, it would not be relevant. The most likely reason is that the parameters of the shockwaves used in this trial are not effective. The energy flux density of 0.3 mJ/mm^2^ in this clinical study was the highest possible unfocused energy flux with this device and has been shown to be effective with diabetic ulcers [19]. We speculate that with a higher local dose, there will be an effect on bone content as recently Shi et al. showed an increase of BMD with the use of focused radial shockwave therapy [31]. As such a focused device only treats a very small area, it is difficult to use this as a therapy to prevent osteoporotic fractures. Unfocused shockwaves, also, might not reach enough depth for the entire bone to be treated [17]. The study of Shi et al. only included osteoporotic patients and treated them accordingly with bisphosphonates, supplemental vitamin D, and calcium, which may explain the differences as well. Previously, we also demonstrated that the use of bisphosphonates in osteoporotic rats induces stronger effects of shockwave therapy [10]. Also, Gerdesmeyer et al. demonstrated that pathologic circumstances show different results of shockwave therapy. They demonstrated that patients with low BMC/BMD were more sensitive to shockwave treatment and increased more bone mass compared with subjects that had a normal or high BMC/BMD [32]. Future research should focus on osteoporotic patients with or without bisphosphonates, and preferably comparing focused and unfocused shockwave therapy. It should also focus on a more potent treatment protocol. One more reason for our negative finding could be that the shockwaves in our study were applied to the distal forearm, where cancellous bone is predominant. Previous bone measurements have shown that shockwave induced bone formation has a more persisted effect on cortical bone [10, 11]. Also, there is a possibility of a type 2 error, in which case we missed a significant difference due to the low study power in the current study. Although the effect of shockwave therapy was negligible in our study, to draw an definite significant conclusion a study is required with a much larger group of 174 patients per group, as was indicated by the power analysis based on our current findings.

A limitation of the use of shockwave therapy for bone regeneration is the use of anesthesia that is required because of UESW-related pain, in particular with the magnitude of the energy level used in the current study. We applied shockwave therapy during a surgery for another indication where aesthesia was required anyway and concluded that shockwave therapy is safe and non-invasive under the current circumstances.

In conclusion, we have shown that a single treatment with unfocused ESW of 0.3 mJ/mm^2^ energy flux is not likely to result in increased BMC or BMD of the forearm. However, to draw a final conclusion, a power analysis indicated that a study with 174 patients per group is required to show an effect size of 0.3 with a power of 80%.
